# Using a Fitbit-based Walking Game to Improve Physical Activity Among U.S. Veterans

**DOI:** 10.1093/milmed/usae280

**Published:** 2024-09-06

**Authors:** Jacob E Simmering, Linnea A Polgreen, Shelby L Francis, Austin J Strom, Alberto M Segre, Philip M Polgreen

**Affiliations:** Department of Internal Medicine, University of Iowa Hospitals and Clinics, Iowa City, IA 52242, USA; Office of Rural Health, Veterans Rural Health Resource Center, Iowa City Veterans Affairs Health Care System, Iowa City, IA 52246, USA; Department of Pharmacy Practice and Science, University of Iowa, Iowa City, IA 52242, USA; Department of Internal Medicine, University of Iowa Hospitals and Clinics, Iowa City, IA 52242, USA; Office of Rural Health, Veterans Rural Health Resource Center, Iowa City Veterans Affairs Health Care System, Iowa City, IA 52246, USA; Department of Computer Science, University of Iowa, Iowa City, IA 52242, USA; Office of Rural Health, Veterans Rural Health Resource Center, Iowa City Veterans Affairs Health Care System, Iowa City, IA 52246, USA; Department of Computer Science, University of Iowa, Iowa City, IA 52242, USA; Department of Internal Medicine, University of Iowa Hospitals and Clinics, Iowa City, IA 52242, USA; Office of Rural Health, Veterans Rural Health Resource Center, Iowa City Veterans Affairs Health Care System, Iowa City, IA 52246, USA

## Abstract

**Introduction:**

Physical inactivity, hereafter inactivity, is a serious health problem among U.S. veterans, hereafter veterans. Inactive adults are at risk for adverse cardiac events and premature mortality. Specifically, among veterans, inactivity has been associated with a 23% increase in mortality. In order to increase physical activity among veterans, we developed Veterans Affairs (VA) MapTrek, a mobile-phone-based web app that allows users to take a virtual walk in interesting locations around the world while tracking their progress against that of others like themselves on an interactive map. Steps are counted by a commercially available Fitbit triaxial accelerometer, and users see their progress along a predefined scenic path overlaid on Google Maps. The objective of this study was to determine the effectiveness of VA MapTrek to increase physical activity in a population of veterans at risk for obesity-related morbidity.

**Materials and Methods:**

We recruited overweight and obese veterans obtaining care at the Iowa City Veterans Affairs Health Center. Half of the veterans were assigned to participate in VA MapTrek. Each week, participants were assigned virtual walking races (Monday through Saturday), which followed a predetermined route that is displayed on Google Maps. The participant’s position on the map is automatically updated each time their Fitbit syncs to their phone. In addition, challenges were issued periodically. Veterans in the control group were only given a Fitbit. We regressed daily step counts on the days of the week, the days since the start of the intervention period, whether the user was in the VA MapTrek or Control group, and an interaction between the study group and the days since the start of the intervention period. We included subject-specific random intercepts and subject-specific random slopes. This model was estimated using Bayesian Hamiltonian Monte Carlo using Stan’s No-U-Turns sampler. We set vague, uniform priors on all the parameters.

**Results:**

We enrolled 276 participants, but only 251 (102 in the control group and 149 in the VA MapTrek group) contributed data during the intervention period. Our analysis suggests an 86.8% likelihood that the VA MapTrek intervention led to a minimum increase of 1,000 daily steps over the 8-week period, compared to the control group. Throughout the 8-week intervention, we project that VA MapTrek participants would have taken an extra 96,627 steps, equivalent to 77.8 additional kilometers (km) (48.3 additional miles), assuming an average of 1,242 steps per km (2,000 steps per mile).

**Conclusions:**

Our study underscores the potential of VA MapTrek as an intervention for promoting walking among veterans who face elevated risks of obesity and cardiac issues. Rural veterans are a high-risk population, and new interventions like VA MapTrek are needed to improve veterans’ health.

## INTRODUCTION

Inactivity is a serious health problem among veterans. In a study of 5,890 male veterans, more than half were physically inactive.^1^ Inactivity is a major risk factor for obesity,^2^ but regardless of body mass, inactivity is a risk for several cardiovascular diseases.^3^ Inactive adults have twice the risk of adverse cardiac events as highly active adults,^4^ and inactivity is associated with 9% of all deaths.^5^ Among veterans, inactivity was associated with a 23% increase in mortality.^1^ The Physical Activity Guidelines for Americans recommend that adults engage in at least 150 to 300 minutes of moderate-intensity physical activity (PA), 75 to 150 minutes of vigorous-intensity PA, or an equivalent combination each week.^6^ However, even moderate amounts of PA, e.g., 30 minutes of walking per day, can facilitate weight loss in overweight and obese individuals.^7^ Activity also helps regulate appetite,^8^ and an increase of 1,000 steps per day has been associated with increased glycemic control in patients with type 2 diabetes (T2D).^9^ Increased time walking is also associated with increased quality of life in all domains, including decreased depressive symptoms.^10^ Given the prevalence of obesity and inactivity among veterans using VA services,^11^ new interventions to help increase levels of PA among U.S. veterans are needed.

The Office of Veterans Affairs (VA) has responded to the inactivity and obesity problems with initiatives to improve health through lifestyle changes. One such response is the MOVE! Weight Management Program and the Whole Health Clinics. The MOVE! Program has been shown to be effective in encouraging weight loss.^12–14^ Another response is the Whole Health Clinic, which focuses on treating well-being in addition to health.^15^ While proving to be successful, these programs require attendance at a VA location and only last for a finite period of time. However, upon program completion, there is an unmet need to provide continued engagement. Ideally, the solution would be to offer a program suitable for long-distance deployment, especially to veterans living in rural areas. This program should also scale to expand in a cost-effective manner to serve veterans in all areas without requiring significant additions of personnel or increasing workloads in existing VA staff.

VA MapTrek, an m-health intervention, is a mobile-phone-based web app that allows users to take a virtual walk in interesting locations around the world while tracking their progress against that of others like themselves on an interactive map.^16–18^ Steps are counted by a commercially available Fitbit triaxial accelerometer, and users see their progress overlaid on Google Maps. Communication occurs via SMS text messaging, a ubiquitous communication medium.^7^ Using familiar technologies makes VA MapTrek easy to use. Beyond Fitbit, it does not require additional apps, logins, or passwords to remember. Registering a Fitbit and mobile phone during one appointment at a VA clinic allows users to play for the entire study. The objective of this study is to determine the effectiveness of VA MapTrek to increase PA in a population of veterans at risk for obesity-related morbidity. Our hypothesis is that veterans randomized to VA MapTrek would walk more than veterans in the control group.

## MATERIALS AND METHODS

### Participants

We recruited English-speaking veterans obtaining care at the Iowa City Veterans Affairs Health Center or at a participating VA Community-Based Outpatient Clinic (participating clinics included Bettendorf, Cedar Rapids, Coralville, Decorah, Dubuque, Ottumwa, and Waterloo, Iowa, as well as Galesburg, Quincy, and Sterling, IL). We recruited veterans who owned a smartphone, were willing to download the Fitbit app onto their phone, had a body mass index (BMI) of at least 25 kg/m^2^, and were able to provide their own written informed consent. Veterans were recruited through their participation with the MOVE! Weight Management Program, the Whole Health Clinic, or by visiting a participating clinic. MOVE! is a weight management and health promotion program designed to improve the lives of veterans by encouraging healthy eating behavior, increasing PA, and promoting weight loss. The Whole Health Clinic approaches care by supporting health and well-being. Research staff met with interested and eligible veterans after a MOVE!, Whole Health Clinic, or other clinic visit to enroll veterans into the study. All study procedures were reviewed by the Iowa City VA Research and Development Office and the University of Iowa Institutional Review Board.

At the initial visit, veterans provided informed consent, completed a baseline survey interview style with research staff, were randomized to either the VA MapTrek group or the control group, and were provided with a wrist-worn Fitbit. The baseline survey asked for demographic information such as race, ethnicity, marital status, blood pressure, and zip code (for urban/rural classification).^19^ Randomization occurred by having each veteran choose from 1 of 2 identical folders: The folders were blank on the outside and were the same thickness. The VA MapTrek folders contained instructions for participating in the VA MapTrek races, which the research staff reviewed with the veteran. The control folders contained blank pages. This method allowed the veterans to understand why they were randomized to a specific group. All veterans were given a Fitbit and taught how to wear and charge it. They were instructed to begin wearing the Fitbit that day and to continue wearing it until the study was complete. They were also told to wear their Fitbit except when showering and swimming and to sync their Fitbit to the Fitbit app to record activity. The days of activity recorded between enrollment and the following Sunday were considered baseline data.

### VA MapTrek Group

Half of the veterans were assigned to participate in VA MapTrek, a text-message-based race game designed to increase step counts previously used in sedentary office workers,^17^ people with obesity or pre-diabetes,^18^ and retirement home residents.^16^ The VA MapTrek participant interface is a platform-independent web app (no app installation necessary). VA MapTrek automatically sends and receives bidirectional text messages to participants via a commercial web-to-short message service gateway (www.twilio.com). Each week, players were assigned to a virtual walking race (Monday through Saturday), which followed a predetermined route displayed on Google Maps. Routes included cities: Istanbul, London, Madrid, New York, Paris, Sydney as well as other scenic areas: The Grand Canyon, The Alps, The Pyrenees, and Denali and Glacier National Parks. Each participant was represented by a chosen icon on a map that advanced along the weekly race route based on data obtained from the Fitbit. Therefore, the more active the participant was in real life, the faster they moved along the virtual race route. The participant’s position on the map was automatically updated each time their Fitbit synced to their phone. Thus, the scenery did not change while the veteran was walking, and there were no simulated changes in altitude when walking in mountainous areas.

Each week, participants were placed into a league with other veteran participants who had a similar activity level during the previous week. The purpose of the activity-based leagues was to maintain strong competition among similarly active participants and minimize discouragement. The first league assignment was determined by baseline step count data.

In addition, challenges were issued periodically. All challenges were based on daily step counts: “Take X steps today to receive X bonus steps toward your race progress.” If a participant completed a challenge, they were awarded bonus steps to move more quickly on the route. Goals ranged from 2,500 to 20,000 steps, based on the performance of each participant, and rewards ranged from 600 to 4,000 steps, with more steps being awarded to higher goals.

### Control Group

The other half of the veterans were given a Fitbit activity tracker but were not enrolled in the VA MapTrek game. They were instructed to do their normal activities for the duration of the study.

Users in the VA MapTrek arm played 8-week-long matches, while control participants were followed for 8 weeks.

## Analysis

### Baseline Measures

A total of 276 veterans were enrolled in the study. At baseline, we recorded demographic (age, sex, race, urban/rural, and marital status) and clinical (blood pressure, BMI, medication use, and comorbidities^20^) information for all participants in the study. Additionally, for each player, we have up to 7 days of baseline step count data. For the demographic and clinical factors, we report simple means, percentages, and standard deviations.

### Step Count Data Processing

Daily summary step count data for each participant during the study period was obtained from the Fitbit application programming interface. We discarded any days with zero steps recorded as non-wear days (*n* = 345 days). We also discarded any days with greater than 50,000 steps (*n* = 7 days or roughly the 99.99th percentile of observed data) recorded as malfunctioning days. We defined compliance as any day with at least 1 but fewer than 50,000 steps.

### Intervention Effect

Our primary interest was whether the intervention increased the daily number of steps taken by participants. We also measured whether the intervention group reduced their daily steps counts by a faster rate following the intervention. We modeled the effect of the intervention using only data after baseline data collection (e.g., the date the intervention started) and going forward for the next 8 weeks. We regressed daily step counts on the days of the week, the days since the start of the intervention period, whether the user was in the VA MapTrek or Control group, and an interaction between the study group and the days since the start of the intervention period. In other words, we assumed the intervention has an immediate effect, captured by the VA MapTrek versus Control indicator, and potentially different rates of step decay between the Control and VA MapTrek groups, captured by the interaction between the study group and days since start of the intervention period. Additionally, there are strong days of the week, especially weekday versus weekend, changes in step counts, and we included these variables to reduce the residual error in the model. Finally, we included subject-specific random intercepts to allow for differences in baseline activity between people and subject-specific random slopes to allow for differences in the rate of decay of the intervention.

We adapted a Bayesian inference approach to our analysis. Unlike frequentist models with analytical solutions, modern Bayesian inference depends on the use of simulation approaches. Our model was estimated using Bayesian Hamiltonian Monte Carlo using Stan’s No-U-Turns sampler (rstan version 2.21). We set vague, uniform priors on all the parameters: The model coefficients as $U\left( { - \infty ,\infty } \right)$, the prior on day-to-day standard deviation in step counts within a person as $U\left( {0;25,000} \right)$ and on the on-diagonal elements of $\Sigma $, the covariance matrix of the subject-specific random intercepts and slopes, as $U\left( {0;25,000} \right)$. Based on prior studies by our group and others, we expected the standard deviation of day-to-day and between-person step counts to be in the range of 3,000 to 5,000 steps per day; 25,000 is 5 times larger than the expected value but imposing this limit significantly speeds up sampling. We placed a Lewandowski, Kurowicka, and Joe prior on the off-diagonal elements of $\Sigma $ with a shape parameter of 2 reflecting our belief that there would be correlation between the subject-specific random effects but still retaining a relatively weak prior.

As Bayesian inference depends on drawing a large number of random samples to estimate the posterior, it is important that we assess whether we have drawn enough random samples to be confident in our inference. We ran 6 chains for 25,000 draws, discarding the first 5,000 draws from each chain as burn-in. These first draws are more likely to be inaccurate, extreme, and not reflect the equilibrium. We assessed the convergence of the 6 chains of 20,000 post-burn-in draws using 3 measures. First, $\hat R$ compares the within-chain variability to the between-chain variability. If all the chains have converged to the same distribution, the within-chain variability and the between-chain variability should be equal, and $\hat R$ should be close to 1.00. Next, we inspected the effective sample size (ESS), which is the effective number of independent draws from the posterior distribution. While we have 6 chains providing 20,000 draws each, each chain is auto-correlated—the current draw value depends on the previous draw’s value. The ESS “adjusts” for this autocorrelation giving us an estimate of the number of independent samples that we have from the posterior distribution for the calculation of means and credible intervals. Lastly, we compare the value of the Monte Carlo error to the total parameter error. The total parameter error comes from 2 sources: Uncertainty in the value of the random variable and uncertainty because of the Monte Carlo estimation procedure. Monte Carlo error is reduced by drawing more samples. If the Monte Carlo error is small (<10% of the total parameter error), this suggests that the observed variability cannot be reduced by drawing more samples. We report the mean of the posterior probability distributions for each parameter as well as the 95% credible intervals.

## RESULTS

### Baseline Characteristics

All our demographic measures (age, sex, urban versus rural, and marital status) were well balanced between the 2 groups as measured by Cohen’s d or Cramer’s V effect sizes (**Table I**). The VA MapTrek group was slightly younger—2 years—than the control group on average. Our clinical measures were also balanced (blood pressure, medication use, and number of comorbidities) although our control group had a slightly higher BMI (33.7 versus 32.9). Common comorbidities included hyperlipidemia (180 participants), hypertension (160), sleep apnea (127), diabetes (83), gastroesophageal reflux (79), and PTSD (57).

**TABLE I.** Study Population Descriptive Statistics and Effect Sizes for Baseline Demographic, Clinical, and Activity Measures

	Control group (*n* = 123)	VA MapTrek group (*n* = 153)	Cohen’s d (continuous) or Cramer’s V (categorical)
**Demographics**			
Age	61.9 (13.0)	59.9 (12.7) [151]	−0.16
Female	12 (9.8)	23 (15.0)	0.05
Urban	55 (45.1) [122]	77 (50.3)	0.00
Race			
Non-Hispanic White	109 (88.6)	132 (86.3)	0.00
Black	4 (3.3)	9 (5.9)	
Hispanic	5 (4.1)	6 (3.9)	
Other	5 (4.1)	6 (3.9)	
Marital status			
Currently married	83 (67.5)	113 (73.9)	0.00
Previously married	29 (23.6)	31 (20.3)	
Never married	4 (3.3)	5 (3.3)	
Unknown	7 (5.7)	4 (2.6)	
**Clinical factors**			
BMI	33.7 (5.8) [121]	32.9 (5.6) [152]	−0.13
Blood pressure			
Systolic	130.0 (14.6) [121]	130.1 (14.6) [152]	0.01
Diastolic	79.1 (9.3) [121]	79.1 (8.2) [152]	0.00
Number of medications	9.4 (5.7)	9.6 (6.9)	0.02
Number of comorbidities	10.3 (5.4)	9.7 (5.9)	0.09
**Baseline step counts**			
Mean days with data	4.6	6.9	–
Average steps per day	6,754 (3,545) [108]	7,023 (4,080) [147]	0.07

Values reported are mean/*n* and (standard deviation/percent). When the sample size differs from the overall (e.g., there are missing data for a variable), the sample size with non-missing data is included in square brackets. The differences between the groups are described using the effect size measures of Cohen’s d (for continuous variables, range [−1, 1]) and Cramer’s V (for categorical variables, range [0, 1]). In both scales, values closer to zero have less imbalance between the groups.

Without adjustment for repeated measures or day-of-week effects, our baseline daily step counts were similar: 6,754 steps per day, on average, among the control participants compared to 7,023 among the future VA MapTrek players (**Table I**). Thus, there is no reason to suspect that randomization failed to balance the groups on relevant observed or unobserved factors.

### Model Results

**FIGURE 1.**
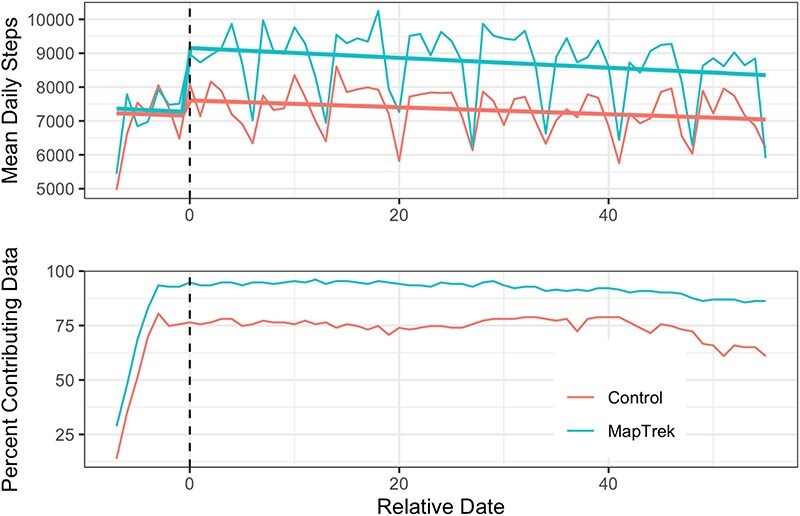
Daily observed step count and Fitbit wear rates by study group. The top panel shows the daily mean step counts for users in each group by relative date (0 = start of intervention period). The 7-day pattern in step counts in both groups during the intervention is because of day-of-week effects. The lower panel shows the percentage of the sample contributing valid data from study group and relative date.

Of the 276 enrolled participants, only 266 participants successfully linked their Fitbit and recorded any data (114/123 in the control group, 152/153 in the VA MapTrek group) during either the baseline or the intervention phase. Only 251 (102 in the control group and 149 in the VA MapTrek group) contributed data during the post-baseline period. There were no significant differences in demographics, comorbidities, medications, BMI, blood pressure, etc., between those who contributed data during the post-baseline period and those who did not. During the post-baseline period, we should have 276 (number of enrolled participants) × 56 days (duration of follow-up) = 15,456 observation days. We have 13,120 observation days during that period (84.9% of the expected number based on the enrolled population and 93.3% of the expected number based on the number who contributed any data during follow-up). Daily percent wearing the Fitbit and contributing valid data as well as daily mean step counts are reported in Fig. 1.

All our convergence measures indicated both sufficient mixing, convergence of the chains, and an adequate number of draws. All our $\hat R$ values were less than 1.01 with nearly all being less than 1.001, suggesting that all 6 chains converged and sampled the complete posterior distribution. Our Monte Carlo standard error was always below 0.7% of the parameter standard error with a mean of 0.3%, suggesting that the randomness due to sampling is only a minor contributor to our parameter error. Additionally, our ESS after adjusting for autocorrelations in draws is very large; always over 23,337 with a mean of 178,000 posterior draws. These results suggest that we have adequately and completely sampled the posterior distribution and have made enough post-burn-in draws to accurately describe the distribution.

Immediately upon starting the intervention, the VA MapTrek group took an average of 1,646 (95% credible interval: 511-2,781) more steps per day and had a non-significantly more rapid decrease in step counts over time (−8.3 steps/day faster decay, 95% credible interval: −26.3 to 9.6; **Table II**). There is a 99.8% posterior probability that the VA MapTrek effect is greater than 0 steps per day at the start of the intervention, 97.6% probability that it exceeds 500 steps per day, and an 86.8% probability that it exceeds 1,000 steps per day. There is an 81.8% posterior probability that the VA MapTrek group loses steps at a faster rate than among the control users over time. However, some participants had a larger-than-average random slope (increase in steps or slower decay over time) and larger-than-average random intercept (more steps per day): 21 MapTrek participants and 9 control participants were in this category.

**TABLE II.** Model Results for Fixed Effects. The Estimates Represent Daily Steps. Veterans in the VA MapTrek Group Took an Average of 1,646 More Steps per Day Than Those in the Control Group at the Beginning of the Study, but This Increase Fell by 8.3 Steps per Day

	Estimate	Std. error	95% credible interval	Monte Carlo Std. error	ESS	$\hat R$
Intercept	5,710	455	4,813-6,606	2.9	25,083	1.000
Days of the week						
Sunday	0.00	Reference				
Monday	2,214	128	1,963-2,464	0.3	138,732	1.000
Tuesday	1,987	129	1,733-2,241	0.3	141,722	1.000
Wednesday	1,979	129	1,726-2,232	0.3	138,540	1.000
Thursday	2,319	129	2,067-2,571	0.3	141,093	1.000
Friday	2,303	128	2,052-2,555	0.3	141,729	1.000
Saturday	1,443	129	1,191-1,695	0.3	136,922	1.000
Days since intervention	−5.4	7.1	−19.4 to 8.5	0.0	62,438	1.000
VA MapTrek group						
Main effect	1,646	578	511-2,781	3.8	23,337	1.000
Interaction with time	−8.3	9.2	−26.3 to 9.6	0.0	58,517	1.000

Reported are the estimated parameters, the standard error of the posterior samples, the observed 95% credible interval, the Monte Carlo standard error, the effective sample size, and $\hat R$ for each of the fixed effects. The Monte Carlo standard error, effective sample size, and $\hat R$ are measures of model convergence.

When the VA MapTrek main effect was positive and the VA MapTrek slope interaction was negative, we calculated the duration of the intervention effect as $Duration = - \frac{{VA{\ }MapTrek{\ }Group{\ }Effect}}{{VA{\ }MapTrek{\ }Slope{\ }Effect}}$. Based on our model estimates, in this population, the VA MapTrek group would take more steps than the control group for an average of 592 days (95% credible interval: 57-2,147).

Over the course of the 8-week intervention, the VA MapTrek players took an average of 96,627 (95% credible interval: 2,605-190,455) additional steps or an average of 77.8 additional km (48.3 additional miles) (95% credible interval: 2.1-153.3 km or 1.3-95.2 miles), assuming 1,242 steps per km (2,000 steps per mile).

The model estimated a between-people baseline activity difference standard deviation of 4,327 (95% credible interval: 3,941-4,757) steps per day, while the rate of decay of steps over time had a between-people standard deviation of 60.8 (95% credible interval: 53.9-68.4) steps per day. These random effects are highly negatively correlated with a correlation of −0.41 (95% credible interval: −0.51, −0.30) in this sample. In other words, the bigger the subject-specific random intercept, the smaller the subject-specific random slope: Those who took more steps initially had smaller step decreases over time. In addition, there is a large day-to-day variability in the number of steps taken with a residual standard deviation, after explaining the time trend, group effect, and subject-specific effects, of 3,913 (95% credible interval: 3,865-3,961) steps per day.

A total of 3,716 challenges were sent to all participants during the course of the study. The challenges issued per participant ranged from 3 to 45 (mean of 24): 1,980 challenges (53.3%) were satisfied.

## DISCUSSION

Our VA MapTrek intervention participants walked more than those in the control group who used a Fitbit alone. Our analysis suggests an 86.8% likelihood that the VA MapTrek intervention led to a minimum increase of 1,000 daily steps over the 8-week period, compared to the control group. Throughout the 8-week intervention, we project that VA MapTrek participants would have taken an extra 96,627 steps, equivalent to 77.8 additional km (48.3 additional miles), assuming an average of 1,242 steps per km (2,000 steps per mile). However, the average number of steps taken by both groups fell over time.

Many PA studies include people who are relatively healthy; for example, many studies have taken place in workplaces,^21,22^ schools,^23^ or among people who have not yet developed disorders associated with obesity.^24^ However, an increasing number of interventions include people who have already developed disease, including cancer,^25^ heart failure,^26^ and diabetes.^27^ Our study included a population of veterans at high risk for future morbidity. Most participants were obese, had an average of 10 comorbidities, and took around 9 medications. In addition, around half of our sample reside in rural areas. Rurality is a risk factor for obesity and inactivity.^28^ People residing in rural locations are less likely to meet exercise guidelines than urban or suburban adults.^29^ Rural residents have less access to gyms, malls, parks, and walking trials.^29,30^ Given our highly rural and high-risk population, the observed 1,000-step increase associated with VA MapTrek is clinically significant, and it aligns with improvements in glycemic control, as evidenced in related research.^9^

Our study participants were overwhelmingly male: 87.3% of participants were male. In contrast, most activity studies average 20% male participation.^31^ In our own PA studies, 1 using Fitbits,^32^ and 3 using MapTrek,^16–18^ we had an average of 23% male participation. Difficulty recruiting men for PA studies has been reported by other investigators.^33^ In addition, recruited males were less likely to enroll, perform baseline evaluations, and participate compared to females.^33^ With low enrollment and participation, it is difficult to determine which types of PA interventions are successful with men.^34^ Although both men and women appear to prefer activities that are close to home and inexpensive, men are reported to prefer activities that they can do alone, and activities that are competitive and occur outdoors.^35^ Accordingly, MapTrek may be suitable for a male population that is difficult to otherwise recruit and retain in physical-activity interventions.

Most PA studies report differences between the control and intervention groups, but not the trajectory of PA.^21^ We found that although MapTrek users took significantly more steps than those in the control group throughout the study, both groups’ steps decreased over time: They took fewer steps each day, on average, and the control group average returned to the baseline level of activity by the end of the study. We estimated that the MapTrek group would return to the baseline level after 592 days. This decay is similar to our previous studies: 8.3 steps per day in this study, 8.96 steps per day in our study of people with obesity,^18^ but less than our study of sedentary office workers, with 20.4 steps per day.^17^

In addition to the trajectory of PA, adherence to interventions is not reported in many PA studies.^21^ Thus, it is difficult to directly compare our observed levels of engagement to that of other studies. However, at the end of the study, approximately 87% of veterans in the MapTrek group were still participating. Our relatively high level of engagement throughout the study period, we think, is attributable to the interactive and game-like nature of VA MapTrek. Indeed, other exercise games have been shown to increase PA.^36^ Specifically, games with multiple players lead to increased activity, even if the other players are strangers.^37^

This study has limitations. First, we lacked complete knowledge of participants’ Fitbit usage throughout the day. An intent-to-treat approach was adopted, incorporating all days with any steps, potentially resulting in an undercount of steps because of partial wear days. This introduces the possibility of bias if such instances varied between groups. Second, the generalizability of our findings to other veteran populations might be limited, particularly because many of our participants were drawn from the MOVE! Program and the Whole Health Clinic, indicating an existing interest in health improvement that might not be representative of all veterans. In addition, social pressure from the MOVE! Program might have increased activity in this study, but this would have affected both groups. Third, the demographic composition of our veteran participants might not accurately mirror the broader U.S. Veteran population, with a significant portion residing in rural areas, identifying as non-Hispanic White, and married. Fourth, the MapTrek and control groups were not equal at the end of the study because enrollment was stopped prematurely because of the Coronvirus disease 2019 (COVID-19) pandemic. This precluded any analysis such as a 2-way, repeated-measures ANOVA. Fifth, this study required the use of a Fitbit. Future studies could collect data directly from the participants’ phones. Finally, not all steps are equal: More-intense exercise is better for health than less-intense exercise,^38,39^ and we cannot determine the intensity of exercise in this study. However, any movement is helpful, especially in patients with obesity and other comorbidities.^7–9^

## CONCLUSION

Despite several limitations, our study underscores the cost-effective potential of VA MapTrek as an intervention for promoting walking among veterans who face elevated risks of obesity and cardiac issues. Rural veterans are a high-risk population, and new interventions like VA MapTrek are needed to improve veterans’ health.

## Data Availability

The data that support the findings of this study are available on request from the corresponding author. All data are freely accessible.
